# The Oleates

**Published:** 1885-09

**Authors:** 


The Oleates. An investigation into their nature and action, by JOHN V. SHOEMAKER, A. M., M. D. Lectures on Dermatology, Jefferson Medical College, &c., &c. Philadelphia: F. A. Davis, 1217 Filbert street. 1885.

This little work is a collection in book form of the writings and the results of the author's experience with these important compounds. Dr. Shoemaker has done much to call the attention of the profession to the value of the oleates, and it is convenient to have such a *resume* in a handy form for reference.

